# Epidemiology of viral acute lower respiratory infections in a community-based cohort of rural north Indian children

**DOI:** 10.7189/jogh.09.010433

**Published:** 2019-06

**Authors:** Anand Krishnan, Rakesh Kumar, Shobha Broor, Giridara Gopal, Siddhartha Saha, Ritvik Amarchand, Avinash Choudekar, Debjani R Purkayastha, Brett Whitaker, Bharti Pandey, Venkatesh Vinayak Narayan, Sushil K Kabra, Vishnubhatla Sreenivas, Marc-Alain Widdowson, Stephen Lindstrom, Kathryn E Lafond, Seema Jain

**Affiliations:** 1 All India Institute of Medical Sciences, New Delhi, India; 2 SGT Medical College, Hospital & Research Institute, Gurgaon, India; 3 Influenza Division, US Centers for Disease Control and Prevention- India country office, New Delhi, India; 4 US Centers for Disease Control and Prevention, Atlanta, Georgia, USA; 5 Division of Global Health Protection, U.S. Centers for Disease Control and Prevention, Nairobi, Kenya

## Abstract

**Background:**

In India, community-based acute lower respiratory infections (ALRI) burden studies are limited, hampering development of prevention and control strategies.

**Methods:**

We surveyed children <10 years old at home weekly from August 2012-August 2014, for cough, sore throat, rhinorrhoea, ear discharge, and shortness of breath. Symptomatic children were assessed for ALRI using World Health Organization definitions. Nasal and throat swabs were obtained from all ALRI cases and asymptomatic controls and tested using polymerase chain reaction for respiratory syncytial virus (RSV), human metapneumovirus (hMPV), parainfluenza viruses (PIV), and influenza viruses (IV). We estimated adjusted odds ratios (aOR) using logistic regression to calculate etiologic fractions (EF). We multiplied agent-specific ALRI incidence rates by EF to calculate the adjusted incidence as episodes per child-year.

**Results:**

ALRI incidence was 0.19 (95% confidence interval (CI) = 0.18-0.20) episode per child-year. Association between virus and ALRI was strongest for RSV (aOR = 15.9; 95% CI = 7.3-34.7; EF = 94%) and least for IV (aOR = 4.6; 95% CI = 2.0-10.6; EF = 78%). Adjusted agent-specific ALRI incidences were RSV (0.03, 95% CI = 0.02-0.03), hMPV (0.02, 95% CI = 0.01-0.02), PIV (0.02, 95% CI = 0.01-0.02), and IV (0.01, 95% CI = 0.01-0.01) episode per child-year.

**Conclusions:**

ALRI among children in rural India was high; RSV was a significant contributor.

Acute lower respiratory infections (ALRI) are the single largest infectious cause of death among children worldwide, with 1-2 million under-five deaths and 12 million hospitalizations globally [1,2]. In India, 17% of all deaths in children <5 years old are due to pneumonia [3]. In a study from India conducted in 2013, the direct cost of an ALRI-associated hospitalization episode was high and estimated to be 34% of the annual per capita income [4]. This high disease and economic burden call for evidence-based public health approaches for prevention and treatment of ALRI including a better understanding of etiology.

Unfortunately, the burden and etiology of acute respiratory infection (ARI) including ALRI in India has not been well studied. In a large systematic review of childhood pneumonia in low and middle-income countries (LMICs), India contributed seven of 200 studies identified, despite bearing 20% of all childhood pneumonia deaths globally [5,6]. A review of Indian studies on ARI among children since 1994 highlighted the lack of community-based studies [7]. Some hospital-based studies on pneumonia etiology have been published recently from India [8,9]; however, hospital-based studies suffer from selection bias due to skewed health care seeking based on gender and socio-economic status, especially in LMICs .

Despite limited information, many public health measures for pneumonia control have been initiated in India starting from case management of pneumonia using community-based health workers in the 1990s. However, persistent treatment gaps, concerns of antibiotic overuse, and availability of newer vaccines have prompted advocacy for a more comprehensive strategy including use of vaccines [10]. *Haemophilus influenzae* type b (Hib) vaccine has been introduced as a part of pentavalent vaccine, and pneumococcal vaccine is currently being rolled out as a pilot project in selected districts of India. Influenza vaccine is currently not part of the national immunization schedule. New vaccines are in various stages of development including those for respiratory syncytial virus (RSV) [11]. Evidence-based decisions on the judicious use of these vaccines require agent-specific community-based disease burden estimates.

We established an Acute Respiratory Infection Surveillance Platform in Ballabgarh, India to assess the burden of ARI and ALRI and associated viral etiologies among a community dwelling open cohort of children <10 years old in rural north India.

## METHODS

The study was conducted in rural Ballabgarh and has been described previously [12]. In brief, all children aged <10 years old during the two-year period who were residents of four study villages were approached; and written informed consent to participate was obtained from their guardian/parent. The study was approved by the Institutional Ethics Committee of All India Institute of Medical Sciences (AIIMS), New Delhi (approval letter number IEC/NP-272/2012) and the Institutional Review Board of the U.S Centers for Disease Control and Prevention (CDC), Atlanta (protocol number 6296).

From the 13^th^ August 2012 to the 9^th^ August 2014, each child was visited weekly at home by trained surveillance workers who asked about ARI defined using the European Centers for Disease Control and Prevention case definition as the presence of cough, sore throat, rhinorrhea, or shortness of breath; and among infants <1-year-old, earache or ear discharge was included [13]. If the symptom started in the past week or had worsened from the previous week, trained nurses obtained a detailed clinical history from an adult caregiver and conducted a physical examination to determine if the child had ALRI as is required under the World Health Organization’s (WHO) Integrated Management of Childhood Illnesses (IMCI) for children <5 years old and Integrated Management of Adolescent and Adult (IMAI) Illness for children 5 to 10 years of age [14,15]. Per these WHO guidelines, ALRI was defined as either “possible serious bacterial infection,” “severe pneumonia or very severe disease” or “pneumonia” while “no pneumonia” were classified as acute upper respiratory infections (AURI) (Appendix S1 in **Online Supplementary Document**) [12,14,15]. No chest auscultation or radiographical confirmation of pneumonia was performed. Nurses provided clinical treatment and referral to all sick children as recommended under national guidelines. If a child was hospitalized or died in the hospital during the study, admission records were reviewed by a study physician to decide whether the cause of hospitalization or death was related to ALRI. All deaths were investigated using a validated verbal autopsy tool by trained supervisors [16]. For each ALRI case, another child of similar age (±six months) from the same village who did not have ARI/ALRI the same week was selected as a control. A random selection of 10% of all children were re-visited by the field supervisors to ascertain the quality of data collection.

Nasal and throat swabs were collected from all ALRI cases and age-matched asymptomatic controls. Specimens were transported on ice in triple sealed containers within 24 hours to the virology laboratory in Delhi and processed according to standard protocols. Specimen aliquots were stored at -80°C until testing was initiated. Total nucleic acid was extracted from each specimen with the Roche LC 2.0 TNA extractor platform using the Roche MagNa Pure LC 2.0 Total Nucleic Acid Isolation kit (Roche Inc., Mannheim, Germany). Real-time reverse transcription polymerase chain reaction (rRT-PCR) was performed using CDC protocols for the detection of the following respiratory viruses: human metapneumovirus (hMPV), influenza viruses (IV), parainfluenza viruses 1-3 (PIV), and RSV. In early 2014, due to unanticipated and unavoidable events, the laboratory was temporarily closed and relocated which hampered testing for up to six months. To reduce the work load on the laboratory, we tested only a random sample of controls from each week and did not test for human rhinoviruses (hRV) in the second year; thus, hRV results are not included in this analysis. However, tested controls were spread evenly across the study period.

Data were entered into a MySQL database and encrypted data was regularly backed up. A new episode of ARI was defined as presence of new symptoms after a symptom-free interval of 7 days. All weekly data on symptoms for each child were linked to delineate each episode of ARI. Children left the cohort once they were 10 years old, migrated out of the study area, died, or if their guardians withdrew consent. Andersen-Gill method of Cox-regression model was used to measure person-time for estimating ARI episodes and person time contribution to allow for recurrent events [17]. Data were arranged to account for recurrent episodes of ARI/ALRI with an assumption that children were not at risk for experiencing a new ARI episode during an ongoing ARI episode. Person-time (child-year) of risk was used as the denominator for the estimation of incidence of ARI, ALRI, hospitalization, and deaths with 95% confidence intervals (CIs) using the normal approximation method and z-test was used for check for significant difference in proportions. Age of the child was taken as the age during the start of the illness event.

We calculated the prevalence of each pathogen among children with ALRI as the proportion of specimens with agent-specific pathogens detected. We constructed multivariable logistic regression models for each virus to estimate the adjusted odds ratio (aOR) with the dependent variable in each model being the child status of asymptomatic control or ALRI case. For the calculation of the aOR, the presence of a virus was considered as the exposure; we compared the odds of a positive detection of a specific virus between ALRI cases and asymptomatic controls, and adjusted for confounders (age, sex, enrollment month/y, and co-detection of any other virus). We used the aOR to calculate the virus-specific etiologic fraction (EF) or the proportion of cases in the exposed population in which the exposure has played a possible causal role in disease development using the equation EF = (aOR–1)/aOR). We did not account for clustering of ALRI events at individual level because overall incidence of viral ALRI was very minimal. The incidence of the event being so low we considered that the clustering effect is likely to be negligible. Agent-specific crude ALRI incidence rates were multiplied by EF to calculate the adjusted ALRI incidence rates. All analyses were conducted in Stata 12 (StataCorp, Texas, USA). Additional analysis with consideration of 14 days symptom free interval for defining a new episode was done to estimate ALRI incidence and etiology specific adjusted ORs.

## RESULTS

**Figure 1:**
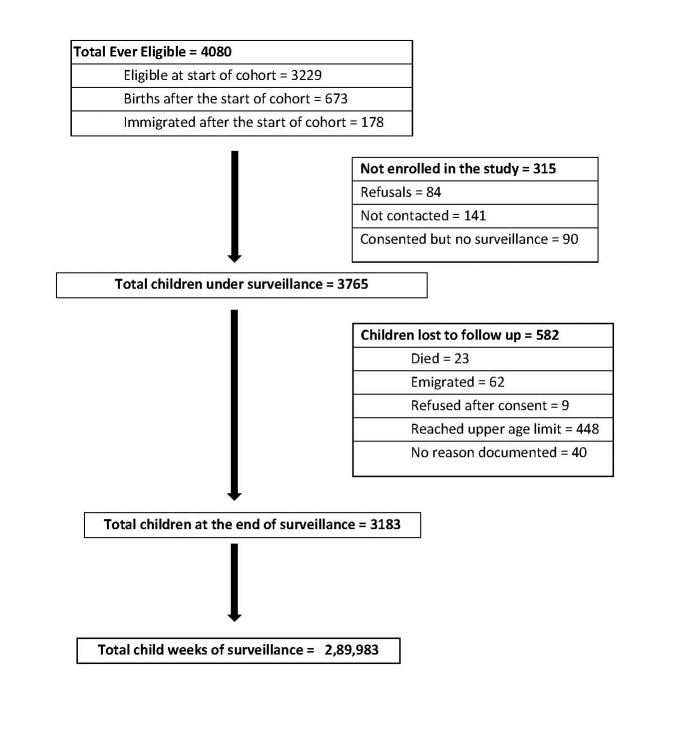
Flowchart depicting the assembly and follow-up of cohort.

A total of 3765 children, 52.8% of which were boys, were followed for a total of 5577 child-years of surveillance contributing to 4666 child-years of risk (**Figure 1**). Attrition in the cohort due to death, migration, and loss to follow-up was 531 child-years and was similar by gender. A total of 29 573 ARI episodes were reported for an overall incidence of 6.3 episodes per child year (95% CI = 6.2-6.4) (**Table 1**). ARI incidence decreased with increasing age, from 10.3 episodes per year (95% CI = 10.0-10.7) among children <1-year-old to 4.4 (95% CI = 4.3-4.5) in children >5 years old. No significant gender differences were noted in ARI incidence. Among all 29 573 ARI episodes, 885 (3.0%) were determined to be ALRI of which 57 (6.4%) resulted in hospitalization and 7 (0.8%) in death. Overall, ALRI incidence was 0.19 episodes per child-year (95% CI = 0.18-0.20); with highest rates among children <1 year old (0.96 episodes per child-year (95% CI = 0.86-1.06)) and children 1-2 years old (0.52 episodes per child-year (95% CI = 0.46 – 0.59)), and lowest rates among children >5 years old (0.03 episodes per child-year (95% CI = 0.02-0.04). Incidence of ALRI was significantly lower among girls than boys, especially in children <3 years old (*P* < 0.05). Three hundred and seventy (9.8%) children had more than one episode of ALRI during the two-year period. One third of ALRI episodes (32.4%) occurred in children <1-year-old. The incidence rate of ALRI reduced to 0.17 episodes/child year (0.16-0.18) when a symptom free period of 14 days was considered for definition of an episode.

**Table 1:** Age and sex-specific incidence (95% confidence interval) of acute respiratory infections (ARI) & acute lower respiratory infections (ALRI) among under-10 children, Ballabgarh, northern India, 2012-14

Age group	Total child-years of follow up	Incidence of ARI (episodes/child year)			Incidence of ALRI (episode/child-years)			Incidence of ALRI-related hospitalization (episode/1000 child-years)		
**Boys**	**Girls**	**Combined**	**Boys**	**Girls**	**Combined**	**Boys**	**Girls**	**Combined**
0-11 mo	359.9	10.8 (10.3-11.2)	9.9 (9.5-10.4)	10.3 (10.0-10.7)	1.14 (1.00-1.30)	0.77 (0.65-0.91)	0.96 (0.86-1.06)	123.0 (79.6-178.8)	46.2 (20.2-89.1)	86.1 (59.2-120.0)
1-2 y	457.3	9.8 (9.4-10.2)	9.2 (8.8-9.6)	9.5 (9.2-9.8)	0.64 (0.55-0.75)	0.39 (0.32 - 0.48)	0.52 (0.46-0.59)	38.5 (17.7-71.7)	13.4 (2.8-38.6)	26.2 (13.6-45.4)
2-3 y	480.9	8.6 (8.3-9.0)	8.3 (7.9-8.6)	8.5 (8.2-8.7)	0.29 (0.23-0.36)	0.16 (0.11-0.21)	0.22 (0.18-0.30)	8.2 (1.0-29.4)	4.2 (0.1-23.2)	6.2 (1.3-18.1)
3-4 y	465.1	8.0 (7.6-8.4)	7.5 (7.1-7.8)	7.7 (7.5-8.0)	0.18 (0.13-0.24)	0.17 (0.12-0.23)	0.17 (0.14-0.22)	17.3 (4.7-43.7)	4.3 (0.1-23.6)	10.7 (3.5-24.9)
4-5 y	482.7	6.6 (6.3-7.0)	6.1 (5.8-6.4)	6.4 (6.2-6.6)	0.11 (0.08-0.16)	0.08 (0.05-0.13)	0.10 (0.07 - 0.13)	3.8 (0.1-21.2)	-	2.1 (0.1-11.5)
<5 y	2245.9	8.6 (8.4-8.8)	8.1 (7.9-8.3)	8.4 (8.3-8.5)	0.43 (0.40-0.47)	0.29 (0.26-0.32)	0.36 (0.34 - 0.39)	33.8 (24.1 - 45.9)	11.9 (6.3-20.3)	23.6 (17.7-30.7)
≥5 y	2420.3	4.4 (4.3-4.6)	4.4 (4.3-4.5)	4.4 (4.3-4.5)	0.03 (0.02-0.04)	0.03 (0.02 - 0.04)	0.03 (0.02-0.04)	1.5 (0.2-5.5)	2.7 (0.6-7.9)	2.1 (0.7-4.8)
**Total**	4666.2	6.4 (6.3-6.5)	6.3 (6.2-6.4)	6.3 (6.2-6.4)	0.22 (0.20-0.24)	0.16 (0.14-0.18)	0.19 (0.18 - 0.20)	16.6 (11.9-22.4)	7.3 (4.2-11.8)	12.2 (9.3-15.8)

ARI – acute respiratory infections, ALRI – acute lower respiratory infections

The overall incidence rate of ALRI-related hospitalization was 12.2 (95% CI = 9.3-15.8) episodes per 1000 child-year (**Table 1**). ALRI hospitalization was approximately twice as high among boys (16.6; 95% CI = 11.9-22.4 episodes per 1000 child-year) as girls (7.3; CI = 4.2-11.8 episodes per 1000 child-year) (*P* < 0.001). Mortality rate due to ALRI in this cohort as determined by verbal autopsy was 1.5 episodes per 1000 child-years (95% CI = 0.2-3.4) and higher among girls (2.2; CI = 0-4.8 episodes per 1000 child-years) than boys (1.4; CI = 0-3.2 episodes 1000 per child-year).

Among the 885 ALRI episodes, 10 episodes did not have swabs collected or had inappropriate samples. Among the 875 ALRI episodes in which swabs were collected, at least one virus was detected in 316 (36.1%) and 19 (2.2%) had more than one virus detected. RSV (16% of ALRI episodes) was the most commonly detected virus and IV (4.8%) were the least commonly detected among the six viruses tested. Nasal/throat swabs were collected from 817 controls but only 500 could be tested due to temporary laboratory problems as described in methods. Among the 500 asymptomatic controls, 30 (6.0%) had at least one virus detected (2 (0.4%) co-detection), including hMPV – 9 (1.8%), PIV – 9 (1.8%), RSV – 7 (1.4%), and influenza – 7 (1.4%).

Each virus was significantly more commonly detected among cases than controls. This association was strongest for RSV (aOR = 15.9; 95% CI = 7.3-34.7) and least for IV (aOR 4.6; 95% CI = 2.0- 10.6) (**Table 2**) resulting in an EF of 94% for RSV and 78% for IV. The adjusted ORs for RSV and HMPV decreased to 15.3 and 6.8 respectively, while for PIV it increased to 7.5 and for influenza the adjusted OR remained unchanged when a symptom free interval of 14 days was considered for definition of a new episode. The adjusted prevalence based on EF among children with ALRI was 14.9% (95% CI = 13.7-15.4) for RSV, 8.7% (95% CI = 7.2-9.4) for PIV, 8.4% (95% CI = 7.0-9.1) for hMPV, and 3.7% (95% CI = 2.4-4.4) for IV. The adjusted ALRI incidence for each virus in episodes per child-year was 0.03 (95% CI = 0.02-0.03) for RSV; 0.02 (95% CI = 0.01-0.02) for hMPV; 0.02 (95% CI = 0.01-0.02) for PIV; and 0.01 (95% CI = 0.01-0.01) for IV (**Table 3**).

**Table 2:** Attributable Etiologic fraction for different viruses in acute lower respiratory infections in children under-ten in rural Ballabgarh, northern India, 2012-14

Pathogen	Total detections among ALRI cases	Prevalence (%) among ALRI cases (95% CI) (n = 875*)	Prevalence (%) among controls (95% CI) (n = 500)	aOR (95% CI)†	Adjusted etiologic fraction % (95% CI)	Adjusted prevalence (%) among ALRI cases (95% CI)
Respiratory syncytial virus (RSV)	139	15.9 (13.5-18.5)	1.4 (0.6-2.9)	15.9 (7.3-34.7)	94 (86-97)	14.9 (13.7-15.4)
Para-influenza viruses (PIV)	88	10.1 (8.1-12.2)	1.8 (0.8-3.4)	7.1 (3.5-14.4)	86 (71-93)	8.7 (7.2-9.4)
Human meta-pneumo virus (hMPV)	86	9.8 (7.9-12.0)	1.8 (0.8-3.4)	7.1 (3.5-14.3)	86 (71-93)	8.4 (7.0-9.1)
Influenza viruses (IV)	42	4.8 (3.5-6.4)	1.4 (0.6-2.9)	4.6 (2.0-10.6)	78 (50-91)	3.7 (2.4-4.4)

ALRI – Acute lower respiratory infection, aOR – Adjusted odds ratio, CI – confidence interval

*10 ALRI episodes did not have swabs collected or had inappropriate sample.

†Adjusted for age, gender, month of illness and viral co-detection.

**Table 3:** Age group and virus-specific adjusted* incidence of acute lower respiratory infection (episodes per child-year, 95% confidence interval), Ballabgarh, northern India, 2012-14

Age group	Child- years of follow up	Respiratory syncytial virus (RSV)	Influenza virus (IV)	Human meta-pneumovirus (hMPV)	Parainfluenza (PI) viruses
0-11 mo	359.9	0.14 (0.11-0.18)	0.02 (0.01-0.04)	0.08 (0.06-0.12)	0.08 (0.06-0.12)
1-2 y	457.3	0.09 (0.06-0.12)	0.02 (0.01-0.04)	0.05 (0.03-0.07)	0.05 (0.04-0.08)
2-3 y	480.9	0.03 (0.02-0.05)	0.01 (0-0.02)	0.01 (0.01-0.03)	0.01 (0.01-0.03)
3-4 y	465.1	0.02 (0.01-0.04)	0.01 (0-0.02)	0.01 (0.01-0.03)	0.02 (0.01-0.04)
4-5 y	482.7	0.02 (0.01-0.04)	0.01 (0-0.02)	0.01 (0-0.02)	0.0 (0.0-0.01)
<5 y	2245.9	0.06 (0.05-0.07)	0.01 (0.01-0.02)	0.03 (0.02-0.04)	0.03 (0.03-0.04)
≥5 y	2420.3	0 (0-0.01)	0 (0-0.01)	0 (0-0.01)	0.0 (0.0 - 0.0)
Total	4666.2	0.03 (0.02-0.03)	0.01 (0.01-0.01)	0.02(0.01-0.02)	0.02(0.01-0.02)

*Adjusted for etiologic fraction derived from **Table 2** – 0.94 for RSV; 0.78 for IV, 0.86 for hMPV, 0.86 for PI.

**Figure 2:**
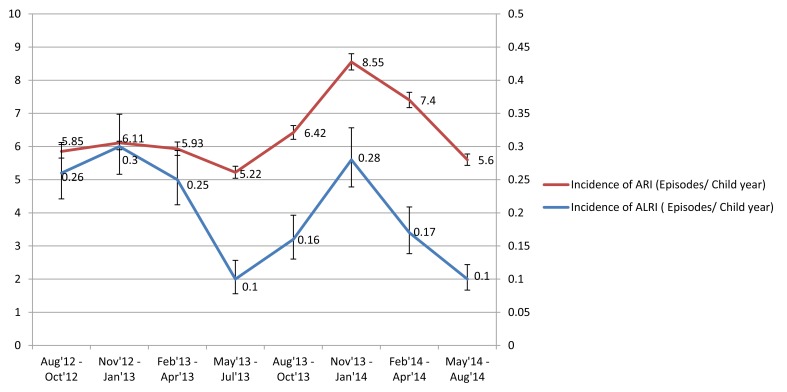
Seasonal variation in incidence rates of acute respiratory infection and acute lower respiratory infection among study children in Ballabgarh, northern India, 2012-14. ARI – Acute respiratory infection, ALRI – Acute lower respiratory infection.

**Figure 3:**
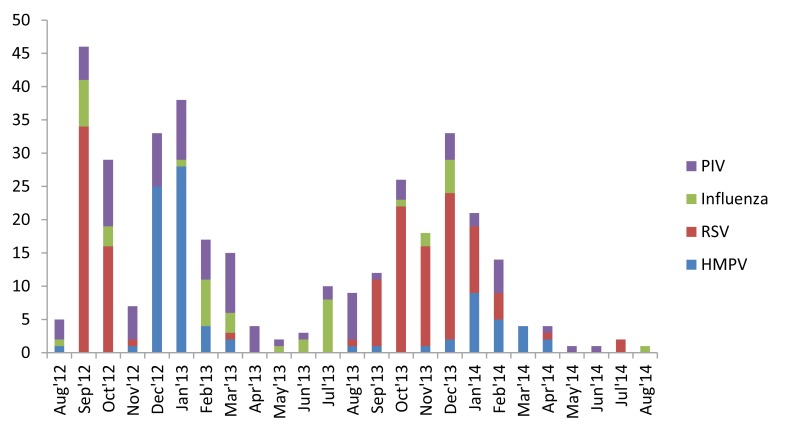
Seasonality of respiratory infections among the study children in Ballabgarh, northern India, 2012-14. hMPV – Human metapneumo virus, RSV – Respiratory syncytial virus, PIV – Para-influenza virus.

Both ARI and ALRI incidence peaked in winters (November-January) and troughed in summers (May-July), before the rainy season during both years of surveillance (**Figure 2**). hMPV demonstrated a clear winter peak (December-January) and RSV usually peaked in the preceding months of September-October. PIV was present throughout the year whereas IV demonstrated varied seasonality during the two years (**Figure 3**).

## DISCUSSION

This is one of the first studies with a large sample size and two years of community-based surveillance to have demonstrated a substantial burden of ARI and ALRI in children <10 years old in rural India. Viruses were significantly more commonly detected among children with ALRI as compared with asymptomatic controls. Our study adds to the evidence base that several viruses, especially RSV, contributed substantially to ALRI in children <5 years old in India and presents data for children 6-10 years old for the first time in India.

Our results demonstrate a high burden of ALRI among children in this rural Indian village in Northern India and are consistent with previously published data from this same area. In our study, we demonstrate an ALRI incidence of 0.53 per child-years in children <3 years old. Broor et al. reported 0.54 episodes per child-year in children <3 years old from 2003-05 in this same study area [18]. We report the ALRI incidence of 0.36 episodes per child-year among children <5 years old which is similar to the global estimates of pediatric community-acquired pneumonia in LMICs for 2010 based on the WHO definition which is approximately 0.22 (IQR; 0.11-0.51) episodes per child-year [19]. Our estimates of ARI incidence of 6.3 episodes per child year among children <5 years old is much higher than 3.6 ARI episodes per child-year reported by Reddaiah et al in 1985-86 [20].

Compared with boys, girls had significantly lower rates of ALRI and ALRI hospitalization in our study. A gender differential among deaths was not found probably due to the small number of deaths in our study. The reasons for these findings are unclear but may be due to a reporting bias where caregivers report or seek care for boys more as compared to girls. An earlier study from this area based on data from 2002-2007 reported higher mortality among girls for diarrhea, prematurity, and malnutrition but not for ALRI [21]. Our concurrent study on measuring number and type of contacts did not show any gender differences in social contact patterns among children <5 years old [22]. Similar ARI incidence rates between genders but not ALRI or subsequent hospitalization indicate that these differences are likely due to differential care seeking. Previous studies from this study area have reported gender discrimination starting before birth in the form of prenatal sex selection and extending to higher use of private facilities and higher expenditure on health care for boys as compared with girls [23,24].

In other studies, conducted in children from neighboring countries, the proportion of ALRI/pneumonia cases with a viral detection has ranged from 40%-98.6%; as compared to 36.1% in our study [25-29]. Meta-analyses conducted by Wang et al. among community-acquired pneumonia (CAP) and Luksic et al. in children hospitalized for pneumonia have estimated the proportion of cases with a virus detection at 57.4% and 50.4%, respectively [29,30]. This estimate is likely to vary depending upon the number of viruses tested; in our study we tested for only 6 viruses and did not include rhinoviruses which could have led to fewer virus detections.

Most studies have identified RSV as a major contributor of hospitalization of children with CAP with proportion ranging from 15.1%-67.0%. For RSV, Wang et al. estimated 17.5% prevalence among children with CAP [30]. In the meta-analysis conducted by Luksic et al. [31], it was estimated that 3% (95% CI = 2.2-4.0) of hospitalized ALRI had IV while Lafond et al. calculated IV prevalence to be between 4%-6% among pediatric hospitalizations globally [32]. Wang et al. calculated a pooled estimate of influenza positivity to be 6.7% (95% CI = 4.7-8.0) among children with CAP as compared to 4.8% in our study [30]. Studies from hospitals in India have also reported similar estimates [9,10]. In an earlier study conducted between 2009 -2011 among hospitalized children in Ballabgarh, RSV was detected in 20%, IV in 7%, and hMPV in 1% of children <5 years old [33]. The wide range of estimates reported from different studies could be due to differences in age groups, settings (ie, hospital, outpatient, community), geographical locations, testing strategy, virus seasonality, case definitions, and study duration. Viral detection among asymptomatic children is uncommon except for hRV [34,35]. In a meta-analysis by Shi et al. to estimate the etiological role of common respiratory viruses in ALRI in children <5 years old, the EF for RSV was 90% and for IV was 80%, as compared with 94% and 78%, respectively in our study. The EF was 70% for PIV and 73% for hMPV in this meta-analysis as compared to 86% for both in our study [36].

Our study has several limitations. We used clinical and syndromic definitions of pneumonia and not radiography which was practical and consistent with what has been done in most community-based studies in LMICs. Pneumonia etiology studies ideally should include specimens from the lung; however, we collected upper respiratory specimens as invasive sampling was not possible in this community. The detection of pathogens in such specimens could represent carriage, infection limited to the upper respiratory tract, or convalescent-phase shedding, and thus detection may not denote causation [37]. Presence of co-infection with >2 pathogens also complicates the issue of attribution of causality. To overcome this, we tested for presence of pathogens among asymptomatic controls to help refine estimates of the attribution to pathogens to ALRI as has been previously applied in pneumonia etiology studies [38,39]. However, more recently based on the Pneumonia Etiology Research for Child Health (PERCH) study, Higdon et al. have proposed that randomly selected community controls, with or without respiratory symptoms, as long as they do not meet the criteria for case-defining pneumonia, are most likely to give an unbiased estimate of etiology. [40] Use of a higher viral load threshold among cases as compared with asymptomatic controls has shown varied results [41,42]. We could have underestimated the burden due to viruses as by the time an ALRI diagnosis is made, children may no longer be shedding the viruses. The controls who were tested were similar in gender to those not tested, but there were differences in age (children aged 2-4 years were less likely to have been tested) and specimens collected later in the study were more likely to be not tested; however, we did adjust for these variables in the equation during the estimation of odds ratio. Given India’s diverse population, studies delineating the epidemiology and etiology of ARI and ALRI in children from other parts of India are needed. Finally, two epidemiological considerations need to be kept in mind while interpreting our incidence rates. First, we used a 7-day symptom-free interval to define a new episode as was performed earlier by Broor et al. [33] in a study in the same area as the definition of an ARI episode is not standardized globally. Second, unlike most studies, the period with ARI was excluded from the denominator time period which reduced the denominator by about 16%.

The results of this study reinforce the need for population-level access to treatment and preventive measures for respiratory infections. Although IV had a lower EF than RSV, it was still a cause of ALRI in this population and as there are influenza vaccines available, including in India, they warrant continued and further study. With multiple RSV candidate vaccines on the horizon, India and other similar LMICs should be considered as sites for evaluation and early implementation to help reduce the burden of ALRI globally. To make evidence-based decisions on use of vaccines, economic evaluations for the introductions of these vaccines into public health programs in India are also needed.

## CONCLUSIONS

The burden of ARI and ALRI is our study was high and RSV was the major contributor.

## Additional material

Online Supplementary Document

## References

[1] Liu L, Oza S, Hogan D, Perin J, Rudan I, Lawn JE (2015). Global, regional, and national causes of child mortality in 2000-13, with projections to inform post-2015 priorities: an updated systematic analysis.. Lancet.

[2] Nair H, Simões EAF, Rudan I, Gessner BD, Azziz-Baumgartner E, Zhang JSF (2013). Global and regional burden of hospital admissions for severe acute lower respiratory infections in young children in 2010: a systematic analysis.. Lancet.

[3] Registrar General of India. Causes of Death in India 2010-2013. New Delhi; 2016. Available: http://www.censusindia.gov.in/2011-Common/Sample_Registration_System.html Accessed: 9 April 2019.

[4] Peasah SK, Purakayastha DR, Koul PA, Dawood FS, Saha S, Amarchand R (2015). The cost of acute respiratory infections in Northern India: a multi-site study.. BMC Public Health.

[5] Gilani Z, Kwong YD, Levine OS, Deloria-Knoll M, Scott JA, O’Brien KL (2012). A literature review and survey of childhood pneumonia etiology studies: 2000-2010.. Clin Infect Dis.

[6] Williams BG, Gouws E, Boschi-Pinto C, Bryce J, Dye C (2002). Estimates of world-wide distribution of child deaths from acute respiratory infections.. Lancet Infect Dis.

[7] Selvaraj K, Chinnakali P, Majumdar A, Krishnan IS (2014). Acute respiratory infections among under-5 children in India: A situational analysis.. J Nat Sci Biol Med.

[8] Singh AK, Jain A, Jain B, Singh KP, Dangi T, Mohan M (2014). Viral aetiology of acute lower respiratory tract illness in hospitalised paediatric patients of a tertiary hospital: one year prospective study.. Indian J Med Microbiol.

[9] Mathew JL, Singhi S, Ray P, Hagel E, Saghafian-Hedengren S, Bansal A (2015). Etiology of community acquired pneumonia among children in India: prospective, cohort study.. J Glob Health.

[10] Ghimire M, Bhattacharya SK, Narain JP (2012). Pneumonia in South-east Asia region: A public Health perspective.. Indian J Med Res.

[11] Mazur NI, Higgins D, Nunes MC, Melero JA, Langedijk AC, Horsley N (2018). The respiratory syncytial virus vaccine landscape: lessons from the graveyard and promising candidates.. Lancet Infect Dis.

[12] Krishnan A, Amarchand R, Gupta V, Lafond KE, Sulliankatchi RA, Saha S (2015). Epidemiology of acute respiratory infections in children - preliminary results of a cohort in a rural north Indian community.. BMC Infect Dis.

[13] European Centre for Disease Prevention and Control. Influenza in Europe – Season 2011–2012.Stockholm: ECDC; 2012.

[14] World Health Organization, Ministry of Health and Family Welfare, Government of India. Integrated Management of Neonatal and Childhood Illness (IMNCI). New Delhi; 2003. Available: https://nhm.gov.in/index1.php?lang=1&level=3&sublinkid=1182&lid=364 Accessed: 9 April 2019.

[15] World Health Organization. Acute care: Integrated Management of Adolescent and Adult Illness. Geneva; 2004. Available: http://applications.emro.who.int/aiecf/web38.pdf Accessed: 9 April 2019.

[16] Kumar R, Kapoor SK, Krishnan A (2012). Performance of cause-specific childhood mortality surveillance by health workers using a short verbal autopsy tool.. WHO South-East Asia J Public Health.

[17] Andersen PK, Gill RD (1982). Cox’s regression model for counting processes: a large sample study.. Ann Stat.

[18] Broor S, Parveen S, Bharaj P, Prasad VS, Srinivasulu KN, Sumanth KM (2007). A prospective three-year cohort study of the epidemiology and virology of acute respiratory infections of children in rural India.. PLoS One.

[19] Rudan I, O’Brien KL, Nair H, Liu L, Theodoratou E, Qazi S (2013). Epidemiology and etiology of childhood pneumonia in 2010: estimates of incidence, severe morbidity, mortality, underlying risk factors and causative pathogens for 192 countries.. J Glob Health.

[20] Reddaiah VP, Kapoor SK (1988). Acute respiratory infections in rural underfives.. Indian J Pediatr.

[21] Krishnan A, Ng N, Kapoor SK, Pandav CS, Byass P (2012). Temporal trends and gender differentials in causes of childhood deaths at Ballabgarh, India - need for revisiting child survival strategies.. BMC Public Health.

[22] Kumar S, Gosain M, Sharma H, Swetts E, Amarchand R, Kumar R (2018). Who interacts with whom? Social mixing insights from a rural population in India.. PLoS One.

[23] Krishnan A, Nawi NG, Byass P, Pandav CS, Kapoor SK (2014). Sex-specific trends in under-five mortality in rural Ballabgarh.. Indian Pediatr.

[24] Selvaraj K, Krishnan A, Gupta SK, Pandav CS (2017). Does gender discrimination transformed its face over few generations? Exploring gender inequalities among under-6 year children in rural Haryana.. Indian J Soc Psychiatry.

[25] Ghafoor A, Nomani NK, Ishaq Z, Zaidi SZ, Anwar F, Burney MI (1990). Diagnoses of acute lower respiratory tract infections in children in Rawalpindi and Islamabad, Pakistan.. Rev Infect Dis.

[26] Ali A, Akhund T, Warraich GJ, Aziz F, Rahman N, Umrani FA (2016). Respiratory viruses associated with severe pneumonia in children under 2 years old in a rural community in Pakistan.. J Med Virol.

[27] Pratheepamornkull T, Ratanakorn W, Samransamruajkit R, Poovorawan Y (2015). Causative agents of severe community acquired viral pneumonia among children in eastern Thailand.. Southeast Asian J Trop Med Public Health.

[28] Homaira N, Luby SP, Petri WA, Vainionpaa R, Rahman M, Hossain K (2012). Incidence of respiratory virus-associated pneumonia in urban poor young children of Dhaka, Bangladesh, 2009-2011.. PLoS One.

[29] Mathisen M, Strand TA, Valentiner-Branth P, Chandyo RK, Basnet S, Sharma BN (2010). Respiratory viruses in Nepalese children with and without pneumonia: a case-control study.. Pediatr Infect Dis J.

[30] Wang M, Cai F, Wu X, Wu T, Su X, Shi Y (2015). Incidence of viral infection detected by PCR and real-time PCR in childhood community-acquired pneumonia: a meta-analysis.. Respirology.

[31] Lukšić I, Kearns PK, Scott F, Rudan I, Campbell H, Nair H (2013). Viral etiology of hospitalized acute lower respiratory infections in children under 5 years of age–a systematic review and meta-analysis.. Croat Med J.

[32] Lafond KE, Nair H, Rasooly MH, Valente F, Booy R, Rahman M (2016). Global Role and Burden of Influenza in Pediatric Respiratory Hospitalizations, 1982-2012: A Systematic Analysis. Global Respiratory Hospitalizations—Influenza Proportion Positive (GRIPP) Working Group.. PLoS Med.

[33] Broor S, Dawood FS, Pandey BG, Saha S, Gupta V, Krishnan A (2014). Rates of respiratory virus-associated hospitalization in children aged <5 years in rural northern India.. J Infect.

[34] Spichak TV, Yatsyshina SB, Katosova LK, Kim SS, Korppi MO (2016). Is the role of rhinoviruses as causative agents of pediatric community-acquired pneumonia over-estimated?. Eur J Pediatr.

[35] Self WH, Williams DJ, Zhu Y, Ampofo K, Pavia AT, Chappell JD (2016). Respiratory viral detection in children and adults: comparing asymptomatic controls and patients with community-acquired pneumonia.. J Infect Dis.

[36] Shi T, McLean K, Campbell H, Nair H (2015). Etiological role of common respiratory viruses in acute lower respiratory infections in children under five years: A systematic review and meta-analysis.. J Glob Health.

[37] Jain S, Pavia AT (2016). Editorial Commentary: The Modern Quest for the “Holy Grail” of Pneumonia Etiology.. Clin Infect Dis.

[38] Bénet T, Sánchez Picot V, Messaoudi M, Chou M, Eap T, Wang J (2017). Microorganisms associated with pneumonia in children <5 years of age in developing and emerging countries: The GABRIEL Pneumonia Multicenter, Prospective, Case-Control Study.. Clin Infect Dis.

[39] Feikin DR, Njenga MK, Bigogo G, Aura B, Aoi A, Audi A (2012). Etiology and Incidence of viral and bacterial acute respiratory illness among older children and adults in rural western Kenya, 2007-2010.. PLoS One.

[40] Higdon MM, Hammitt LL, Deloria-Knoll M, Baggett HC, Brooks WA, Howie SRC (2017). Should controls with respiratory symptoms be excluded from case-control studies of pneumonia etiology? Reflections from the PERCH Study.. Clin Infect Dis.

[41] Feikin DR, Fu W, Park DE, Shi Q, Hingdon MM, Baggett HC (2017). Is higher viral load in the upper respiratory tract associated with severe pneumonia? Findings from the PERCH Study.. Clin Infect Dis.

[42] Fuller JA, Njenga MK, Bigogo G, Aura B, Ope MO, Nderuty L (2013). Association of the CT values of real-time PCR of viral upper respiratory tract infection with clinical severity, Kenya.. J Med Virol.

