# Supplementary material for: Epidemiology of viral acute lower respiratory infections in a community-based cohort of rural north Indian children
Source: J Glob Health. 2019 May 12;9(1):010433. doi: 10.7189/jogh.09.010433 (PMC6513504; doi:10.7189/jogh.09.010433)
Supplement: Online Supplementary Document [file jogh-09-010433-s001.pdf]

J Glob Health 2019; 9 010433

Appendix S1

Case definitions for AURI and ALRI

Clinical signs and symptoms	IMNCI/ IMAI Case definition	Study definition
**Age <2months**		
**Any one of the following**<br>Convulsions **OR**<br>Not feeding well **OR**<br>Fast breathing (60 breaths per minute or more) **OR**<br>Severe chest indrawing **OR**<br>Grunting **OR**<br>Fever (37.5 C* or above) **OR**<br>Low body temperature (less than 35.5 C*) **OR**<br>Movements only when stimulated or no movements even when stimulated	**Possible Serious Bacterial Infection**	**ALRI**
Insufficient signs for the above classification		**AURI**
**Age 2months - 59months**		
Any general danger sign (lethargic/unconscious/convulsion) **OR**<br>Stridor in calm child **OR** Chest in-drawing	Severe Pneumonia or Very Severe Disease	**ALRI**
Fast breathing<br>(2-12 months: 50 breaths per minute or more<br>12 months-59 months: 40 breaths or more)	Pneumonia	**ALRI**
No signs of pneumonia or very severe disease	No Pneumonia: Cough or Cold	**AURI**
**Age 5 years – 10 years**		
**Any one or more of the following**<br>Fast breathing (40 breaths per minute or more) **OR**<br>High fever (38˚C or above) **OR**<br>Pulse rate (≥ 120 per minute) **OR**<br>Lethargy **OR**<br>Severe chest pain	Severe Pneumonia or Very Severe Disease	**ALRI**
**Two of the following**<br>Fast breathing (30 breaths per minute or more) **OR**<br>Night sweats **OR**<br>Chest pain	Pneumonia	**ALRI**

Insufficient signs for the above classification	No pneumonia: Cough or cold	**AURI**
